# Voltammetric Behavior and Determination of Trace Amounts of Omeprazole Using an Edge-plane Pyrolytic Graphite Electrode

**Published:** 2015

**Authors:** Saeed Shahrokhian, Masoumeh Ghalkhani, Maryam Bayat, Fatemeh Ghorbani-Bidkorbeh

**Affiliations:** a *Department of Chemistry, Sharif University of Technology, Tehran, Iran .*; b *Institute for Nanoscience and Technology, Sharif University of Technology, Tehran, Iran .*; c *Department of Chemistry, Faculty of Science, Shahid Rajaee Teacher Training University, Lavizan, Tehran, Iran.*; d *Department of Pharmaceutics, School of Pharmacy, Shahid Beheshti University of Medical Sciences, Tehran, Iran.*

**Keywords:** Omeprazole, Pyrolytic graphite electrode, Voltammetry, Pharmaceutical analysis

## Abstract

The voltammetric performance of edge-plane pyrolytic graphite (EPG) electrode via adsorptive stripping voltammetry was investigated for study of the electrochemical behavior of omeprazole (OMZ) in aqueous solution. The results revealed that the oxidation of OMZ is an irreversible pH-dependent process that proceeds with the transfer of one electron and one proton in an adsorption-controlled mechanism. The determination conditions, such as the pH values of the supporting electrolyte, accumulation time and scan rate were optimized. Simplicity, high reproducibility and low detection limit (3 nM) of the electrode response as well as wide linear range (0.01 to 4.0 µM) can be stated as significant features of this electrode. The EPG electrode was successfully applied for the determination of OMZ in pharmaceutical formulations and satisfactory results were obtained.

## Introduction

Omeprazole (OMZ), a substituted benzimidazole compound and prototype anti-secretary agent, belongs to a new class of potent and clinically active inhibitors of gastric acid secretion (1). OMZ which inhibits the H^+^, K^+^-ATPase, a proton pump that is peculiar to the gastric parietal cell, is widely used in the treatment of gastroesophageal reflux disease. Furthermore it can be used to promote healing of erosive esophagitis and for Helicobacter pylori eradication to reduce the risk of duodenal ulcer recurrence (2-4).

The methods available for the determination of OMZ are mainly based on chromatographic and spectrophotometric procedures. Some of the them are liquid chromatography (LC) (5), liquid chromatography–mass spectrometry (LC-MS) (6), liquid chromatography–mass spectrometry/mass spectrometry (LC-MS/MS) (7), packed column supercritical fluid chromatography (PSFC) (8), hydrophilic interaction liquid chromatography (HILIC) (9), HILIC with tandem mass spectrometry (HILIC-MS/MS) (10), fluorescence spectrophotometery (11) and high performance liquid chromatography (HPLC) with coulometric detection (12). However, these methods suffer from some disadvantages such as high costs, long analysis times and requirement for complex and tedious sample pretreatment, and in some cases, low sensitivity and selectivity that makes them unsuitable for a routine analysis. 

Due to their high sensitivity, electroanalytical techniques have been widely applied for the determination of a wide range of pharmaceutical compounds (13-18). Moreover, investigation of the redox behaviour of drugs and biomolecules by means of electrochemical techniques has the potential for providing valuable insights into the redox reactions of these compounds and might have profound effects on our understanding of their *in-vivo *redox behaviour or pharmaceutical activity. The development of voltammetric sensors for determination of OMZ in pharmaceuticals and clinical analysis has received considerable interest in recent years. Mercury electrode (19-21), carbon paste electrode (22), and glassy carbon electrode (23, 24) have mainly been employed in these researches. However, the electrochemical reaction of OMZ, same as most pharmaceutical compounds, at the surface of bare electrodes involves slow electron transfer kinetics.

Pyrolytic graphite (PG) is one of the most popular electrode materials. Superior to glassy carbon electrodes, it is easy to refresh the surface of PG electrode and its edge sites are more reactive to adsorption and chemical modification, so PG is a promising material for electroanalytical applications (25-27). Edge plane pyrolytic graphite (EPG) is advantageous for use as electrode due to its highly reactive edge plane sites which allow low detection limits, high sensitivities, improved signal to noise characteristics and low over potentials. In many cases EPGE can conveniently replace carbon nanotube-modified electrodes in this area due to their simplicity of preparation, cost, and relative advantages of reactivity (28).

In this work, for the first time, we used EPG electrode in order to trace determination of OMZ. The proposed electrode represents a simple, rapid and sensitive voltammetric method for determination of OMZ.

## Experimental

*Chemicals and reagents*

OMZ (>99.0 % purity) was gently donated by TEMAD Pharmaceutical Co. (Tehran, Iran). Capsules of OMZ for testing analytical application of the method were purchased from Daroupakhsh Co. (Tehran, Iran). All other chemicals and reagents used in this work were of analytical grade from Merck. Voltammetric experiments were carried out in the deoxygenated buffered solutions of OMZ by purging the pure nitrogen (99.999% from Roham Gas Company). A 0.1 M acetate solution was used for the preparation of the buffer solutions with pHs 4 and 5, and 0.1 M phosphate solution for other pH values. All aqueous solutions were prepared with doubly distilled deionized water over the dilute alkaline permanganate solutions.

*Apparatus*

Voltammetric experiments were performed using a Metrohm potentiostat/galvanostat model 757VA. A conventional three-electrode system was used with an EPG electrode of geometric area 0.75391 cm^2^ (Le Carbone, *Ltd*., Sussex, U.K.), as the working electrode, a saturated Ag/AgCl reference electrode and a Pt wire counter electrode. In each measurement, the working electrode was used after an electrochemical cleaning in a background solution of 0.1 M phosphate buffer solution with pH 7.0 by means of one potential sweep at 100 mV s^-1^ within the potential window of -2.0 to +2.0 V (vs. saturated Ag/AgCl reference electrode). Furthermore, before each measurement in a studied solution, the baseline current of the electrode became steady by three times scanning the potential between 0.0 and 1.0 V (scan rate 100 mV s^-1^) in the same buffer solution. All experiments were performed at 25 (±1) ^o^C. A digital pH/^0^C/mV meter (Metrohm, model 827 pH Lab) was used for preparation of the buffer solutions. 

##  Results and Discussion

*Electrochemical behavior of OMZ at EPG electrode *

**Figure 1:**
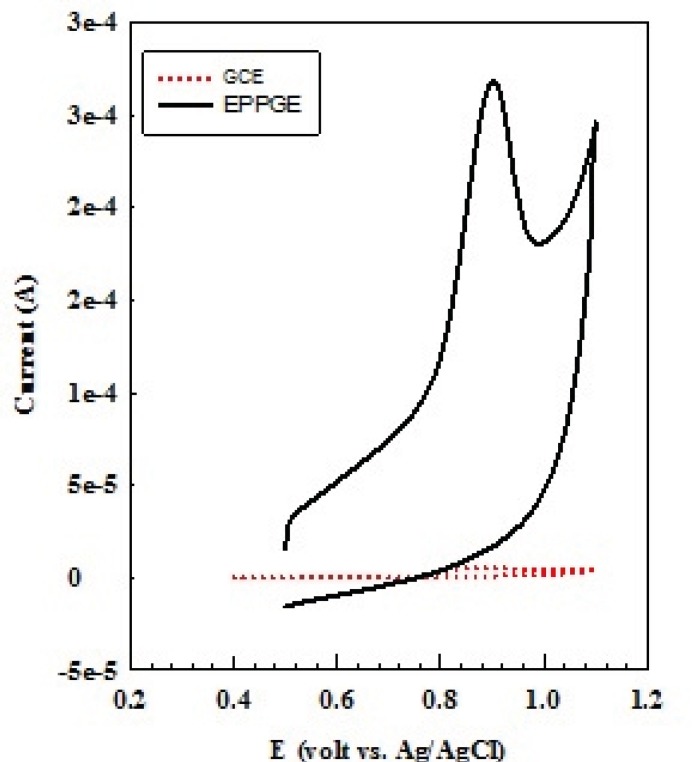
CVs of 100 μM of OMZ in 0.1 M phosphate buffer solution (pH 7.0) at the surface of EPG (solid line) and GC (dotted line) electrodes) scan rate 100 mV s^-1^.

Figure 1 shows voltammetric behavior of 10^-4^ M OMZ at the EPG electrode and glassy carbon electrode (GCE), recorded in 0.1 M phosphate buffer solution at pH 7.0 at the scan rate of 100 mV s^-1^. As can be seen, on scanning from 0.5 to 1.1 V, a well-defined and sharp oxidation peak was obtained for OMZ (solid line) at 0.9 V. Furthermore, no cathodic peak was resulted for OMZ during the reverse scan, suggesting a totally irreversible behavior for the electrode process. On the surface of GCE, the response of OMZ appeared weak and broad (dotted line) due to sluggish electron transfer, while the peak current considerably improved at the EPG electrode. A good response with high oxidation peak current obtained for OMZ at the EPG electrode reflects the excellent electronic conductivity of the edge sites of pyrolytic graphite and the high electroactive surface area of the EPG electrode, which will cause improvement in the determination sensitivity.

The results showed that electrode passivation occurs after several measurements and causes a little reduction in peak current. It is due to the adsorption of oxidation product of OMZ, which are electrochemically inactive over the used potential window. For removing these contaminations from the electrode surface, an electrochemically cleaning step in buffer solution was done before every measurement, as described in previous section.

*The pH effect*

**Figure 2:**
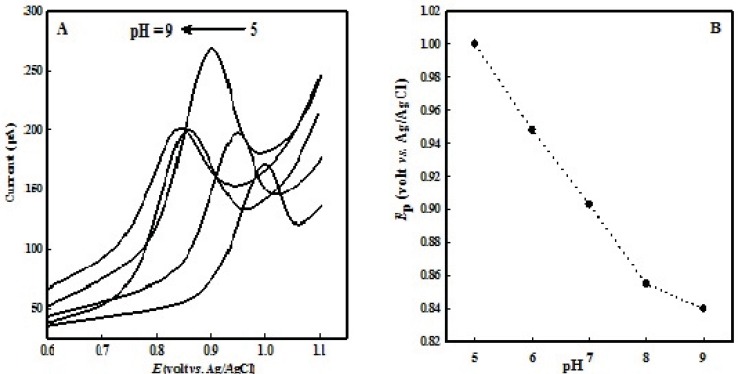
(A) LSVs of 100  M OMZ at the EPG electrode in various pHs of buffer solution (from 1 to 5: 5.0, 6.0, 7.0, 8.0, 9.0). (B) Dependence of *E*_p_ with pH solution; scan rate 100 mV s^-1^.

The organic electrode reaction systems always involve the proton transfer, so a buffer solution is often used as the supporting electrolyte, and the pH of the buffer solution has a direct impact on the peak shape and *E*_1__/__2_. Therefore, pH of buffer solution is very important. OMZ degrades in acidic solutions unless it may be protected against acid conditions (29), so a significant decomposition is observed in solutions with pH values below 5.0 with a color change of solution, so we studied solutions with pH values equal or more than 5.0. Voltammetric behavior of OMZ in buffer solutions with different pHs (from 5.0 to 9.0) was investigated on the surface of the EPG electrode (Figure 2A). A good linear relationship was observed between the *E*_p_ and pH values (in the range of 5 to 8, Figure 2B) with the equation, *E*_p_ (mV) = - 48.0 pH + 1238.5 (R² = 0.9992). It could be observed that the oxidation peak potential shifted negatively with increasing pH, which suggests that H^+^ participates in the oxidation process and OMZ oxidation involves the same number of electrons and protons. In solutions with a pH value greater than 8.0, the slope is changed because the mechanism itself changes to where there are no protons involved before the rate determining step. The best electrode response is achieved in buffer solutions with pH 7.0. Therefore, phosphate buffer with pH 7.0 was chosen as supporting electrolyte for further experiments. 

**Scheme 1:**
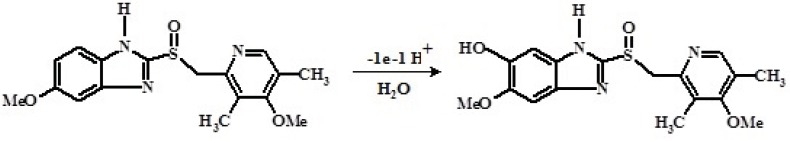
Proposed electrooxidation mechanism of OMZ.

As reported previously (24), the electrochemical oxidation of OMZ involves removal of one electron, followed by deprotonation to form a cationic radical intermediate, which reacts with water and leads to the irreversible production of hydroxylated product. Obtained results are in agreement with proposed oxidation mechanism of OMZ in the literature (24), as presented in Scheme 1.

*The Effect of potential scan rate*

**Figure 3:**
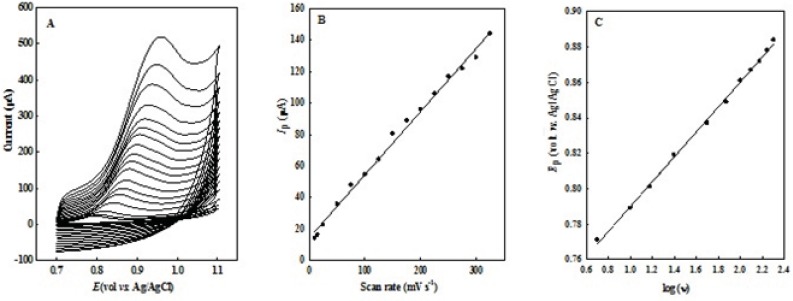
(A) CVs of 100  M OMZ at the EPG electrode at different scan rates (down to up: 10 to 400 mV s^-1^) in 0.1 M phosphate buffer solution (pH 7.0). (B) The plot of *I*_p_ *vs.* potential scan rate, and (C) variation of peak potential (*E*_p_) with log (ν).

The effect of sweep rate (ν) on peak potential (*E*_p_) and peak current (*I*_p_) of 0.1 mM OMZ in the range of 10 - 400 mV s^-1^ in pH 7.0 phosphate buffer solution was studied at EPG electrode (Figure 3). The peak current was directly proportional to the scan rate (ν), *I*_p_= 0.4019 υ + 14.336 (R^2^=0.994, *I*_p_: µA, ν: mV s^-1^), (in the range of 10 - 325 mV s^-1^) with a correlation coefficient (R^2^) of 0.994 (Figure 3B), which suggested an adsorption-controlled process on the surface of the EPG electrode. 

No cathodic peak was observed on the reverse scan at all the potential sweep rates which were studied, representing a totally irreversible process for the electro-oxidation of OMZ. On the other hand, the peak potential (*E*_p_) shifted in positive direction with increasing scan rate. There is a linear relationship between *E*_p_ and logarithm of the scan rate (*E*_p_ = 70.37 log ν + 719.51, R^2^=0.998, *E*_p_: mV, ν: mV s^-1^) (Fig. 3C), which further reveals the irreversible nature of the electrochemical process. 

This behavior is in conformity with the equation for irreversible electrochemical processes (30), *E*_p_= (1/2) b log ν + a (b=2.303RT/(1-α) n_a_F). Here, α is the transfer coefficient, n_a_ the number of electrons involved in the rate-determining step of the electrode process, F the Faraday constant, R the gas constant and T is the absolute temperature. 

The Tafel plot and its corresponding slope were used for elucidating the mechanism of the electrode process. The polarization curves (log *I* vs. *E *plots) for the electro-oxidation of OMZ on the surface of the EPG electrode obtained at various potential sweep rates (25-100 mV s^-1^). The slopes of Tafel plots, ((1- )n_a_ F/2.3RT), show values between 7 and 8.3 for potential sweep rates in the range of 25-100 mV s^−1^. By considering n_a_=1, the value of α was calculated to be in the range of 0.51–0.59, which indicates that the activation free energy curve is not symmetrical in such an irreversible process. 

*Effect of accumulation time*

**Figure 4:**
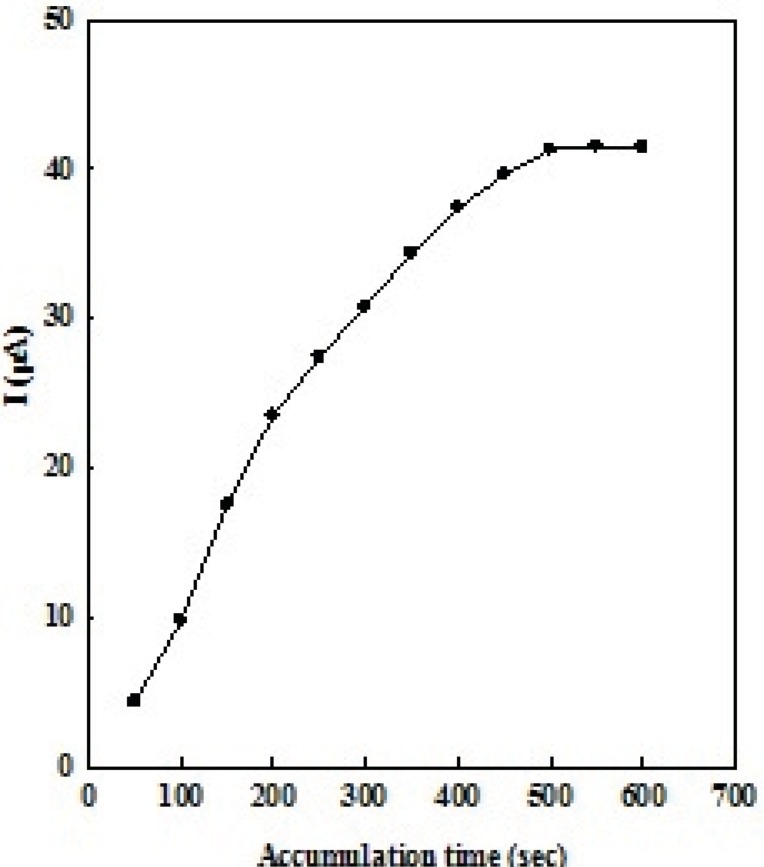
The plot of peak current (μA) versus accumulation time (s) of 1 μM of OMZ at the EPG electrode in 0.1 M phosphate buffer solution (pH 7.0).

Accumulation step is usually a simple and effective way to enhance the sensitivity of the determination. Upon OMZ adsorption on the EPG electrode, application of a preconcentration step to increase the electrochemical response and also the sensitivity is necessary. Therefore, the accumulation of OMZ on the electrode surface, under open circuit, was performed before electrochemical measurements. As for any technique employing preconcentration step, the accumulation time is of significant importance for the voltammetric signal. Results show that the longer accumulation times to 500 s, increase the amount of adsorbed OMZ and therefore give higher peak current. Then, the peak currents remain constant due to the surface saturation (Figure 4). As the OMZ concentration decreases, it takes a longer accumulation time to reach the saturation peak current. A smaller peak current is obtained when a shorter accumulation time is used. A comprise between sensitivity and analysis time has to be considered. Taking account of sensitivity and efficiency and also response repeatability, the accumulation time was set at 500 s as a reasonable comprise in the analytical measurements.

*Analytical measurements*

**Figure 5:**
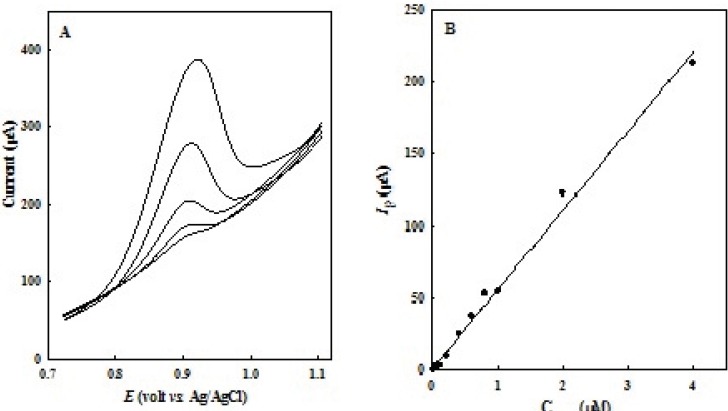
(A) LSVs for various concentrations of OMZ in the range of (down to up), 0.2 to 4.0 μM in 0.1 M phosphate buffer solution (pH 7.0). (B) Corresponding linear calibration curve of *I*_p_ *vs.* OMZ concentration.

LSV peak currents of OMZ increase linearly with the concentration. To verify the linear relationship between anodic peak current and OMZ concentration, a calibration curve was constructed under the optimized experimental conditions in 0.1 M of phosphate buffer solutions (pH 7.0). Figure 5A shows LSVs obtained at EPG electrode in various concentrations of OMZ. A linear dynamic range from 0.01 to 4.0 μM, with a calibration equation of *I*_p_ (μA) = 55.08 C_OMZ _(μM) + 1.127 (*R*^2^ = 0.993), and a detection limit of 3 nM (*S*/*N*= 3) was obtained (Figure 5B). 

The main advantage of using the EPG electrode is easy and quick surface renewal after each use. The repeatability of the EPG electrode was investigated by repetitive detection of 1 µM OMZ at the same electrode, refreshed after each measurement. Indeed, the relative standard deviation (R.S.D.) of 2.8 %, in six consecutive determinations, indicates excellent repeatability of the response of the EPG electrode.

*Analytical application*

Applicability of the EPG electrode was examined for the determination of OMZ in pharmaceutical capsules. The appropriate amount of OMZ capsule solution was spiked with different amount of OMZ standard solution (in the range of 0.1 to 4 M) and its LSV was recorded using EPG electrode at optimum conditions as described earlier. The calibration curve of the peak current versus the concentration exhibited a good linear range. In these measurements, a slope of 52.15 A/M with a correlation (R^2^) of 0.995 was obtained in the same concentration range, showing a good agreement with the slope of calibration curve of standard solutions of OMZ. Such results emphasize very good recoveries for the determinations of OMZ in pharmaceutical capsules and confirm that the proposed method could be efficiently used for the determination of trace amounts of this compound in pharmaceutical preparations. 

## Conclusions

In the present work, the EPG electrode was used to investigate the electrochemical behavior of OMZ. The oxidation of OMZ is an irreversible, adsorption -controlled, pH dependent process, which involves a one electron and one proton transfer to yield a hydroxylated product. Under optimized conditions, a linear dynamic range of 0.01 to 4.0 µM with a detection limit of 3 nM OMZ was obtained. The proposed method has been practically and successfully applied for determination of OMZ in pharmaceutical preparations. The low detection limit, simple cleaning procedure, reproducibility, wide linear dynamic range, high sensitivity and simplicity suggest that this electrode is an attractive candidate as a sensor for practical applications.
